# Population Structure and Selection Signatures in Chinese Indigenous Zhaotong Pigs Revealed by Whole-Genome Resequencing

**DOI:** 10.3390/ani14213129

**Published:** 2024-10-30

**Authors:** Yixuan Zhu, Xiaoyi Wang, Yongli Yang, Lixing Wang, Chengliang Xu, Wenkun Xu, Qiang Chen, Mingli Li, Shaoxiong Lu

**Affiliations:** 1 Faculty of Animal Science and Technology, Yunnan Agricultural University, Kunming 650201, China; 2 Yunnan Provincial Livestock Station, Kunming 650506, China

**Keywords:** Zhaotong pig, Asian wild boar, population structure, selection signature, whole-genome resequencing

## Abstract

Zhaotong pig (ZTP) is a Chinese indigenous pig breed in Yunnan Province, known for its unique body shape and appearance, good meat quality, strong foraging ability, and adaptability. However, there is still a lack of research on its genome. In order to investigate the genetic diversity, population structure, and selection signatures of the breed, we conducted a comprehensive analysis by resequencing on 30 ZTPs and comparing them with genomic data from 10 Asian wild boars (AWBs). A total of 45,514,452 autosomal SNPs were detected in the 40 pigs, and 23,649,650 SNPs were retained for further analysis after filtering. The H_E_, H_O_, P_N_, MAF, π, and Fis values were calculated to evaluate the genetic diversity, and the results showed that ZTPs had higher genetic diversity and lower inbreeding coefficient compared with AWBs. Population structure was analyzed using NJ tree, PCA, ADMIXTURE, and LD methods. It was found that ZTPs were population independent of AWBs and had a lower LD decay compared to AWBs. Moreover, the results of the IBS genetic distance and G matrix showed that most of the individuals had large genetic distances and distant genetic relationships in ZTPs. Selection signatures were detected between ZTPs and AWBs by using two methods, F_ST_ and π ratio. Totals of 1104 selected regions and 275 candidate genes were identified. Finally, functional enrichment analysis identified some annotated genes that might affect fat deposition (*NPY1R*, *NPY5R*, and *NMU*), reproduction (*COL3A1*, *COL5A2*, *GLRB*, *TAC3*, and *MAP3K12*), growth (*STAT6* and *SQOR*), tooth development (*AMBN*, *ENAM*, and *ODAM*), and immune response (*MBL2*, *IL1A*, and *DNAJA3*). Our results will provide a valuable basis for the future effective protection, breeding, and utilization of ZTPs.

## Simple Summary

The purpose of this study was to conduct whole-genome resequencing on Zhaotong pigs and integrate the genomic data of Asian wild boars to evaluate their genetic diversity, population structure, and selection signatures. A total of 275 genes were annotated as candidate genes, some of which might be associated with fat deposition, reproduction, growth, tooth development, and immune response. The findings revealed the genomic information and would contribute to optimizing conservation and breeding strategies of Zhaotong pigs.

## 1. Introduction

As is well known, the pig is one of the earliest domesticated animals. During the domestication process, rich phenotypic diversities have emerged among different pig breeds, due to the differences in preferences for pig breed characteristics in various regions. After a long period of natural selection and strong artificial selection, China has cultivated a large number of indigenous pig breeds with different characteristics, accounting for about one-third of the world’s pig breed resources [1]. However, the genetic diversity and population size of Chinese indigenous pig breeds have been declining, owing to the continuous and large-scale introduction of Western pig breeds and crossbreeding for commercial interests [2,3]. Consequently, it has become necessary to evaluate the genetic diversity and population structure of Chinese indigenous pig breeds to develop more effective conservation strategies and prevent further genetic loss in these breeds.

Currently, with the rapid development of sequencing technology and the decrease in the cost of sequencing, whole-genome resequencing has been used increasingly to study the genetic diversity and selection signature of pigs [1,4,5,6,7]. A large number of candidate genes and genetic markers associated with adaptive, phenotypic, and important economic traits have been identified through genome-level studies. These findings not only contribute to a deep understanding of the origin, domestication mechanism, and selection but also provide an important basis for the genetic improvement of pigs.

Zhaotong pig (ZTP) is a valuable Chinese indigenous pig breed that is mainly distributed in Zhaotong City, Yunnan Province. The unique ecological and geographical conditions, along with the traditional ethnic culture, have enabled ZTPs to adapt to the local environmental condition and exhibit distinctive characteristics, such as unique body shape and appearance, young age of sexual maturity, high proportion of intramuscular fat (IMF), and strong foraging ability and adaptability [8]. As we know, ZTPs also have some obvious shortages similar to most Chinese indigenous pig breeds, such as slower growth and lower carcass lean percentage [9]. In recent years, the main producing area of ZTPs has been gradually shrinking, and the number of pigs has been decreasing year by year due to the promotion of hybrid utilization of pig breeds. Therefore, it is crucial to enhance the protection and utilization of ZTPs. But up to now, research on ZTPs is still rare. Especially, there has been no investigation into the genome-wide genetic characterization of ZTPs. The genetic diversity at the genomic level and the molecular genetic basis of the specific characters of ZTPs are still unclear.

It can help us to understand the effects of domestication on genetic diversity and to identify selected genes or genomic regions during the domestication and selection by comparing indigenous pig breeds with wild boars. In order to investigate the genetic diversity, population structure, and selection signatures of ZTPs, exploring the molecular genetic mechanism of its characteristic traits, we conducted whole-genome resequencing on ZTPs and integrated the genomic data of Asian wild boars (AWBs) to investigate the population structure and selection signatures. Single nucleotide polymorphisms (SNPs) were detected to analyze the population structure, including neighbor-joining (NJ) tree construction, principal component analysis (PCA), ADMIXTURE, and linkage disequilibrium (LD) analysis. Moreover, the fixation index (F_ST_) and nucleotide diversity (π) ratio methods were used to detect selection signatures and annotate the candidate genes to identify genes associated with important economic traits.

## 2. Materials and Methods

### 2.1. Ethics Statement

All of the animals were treated according to China’s national standard of Guidelines on Welfare and Ethical Review for Laboratory Animals (GB/T 35892-2018) [10]. This study was approved by the Ethics Committee of Yunnan Agricultural University (YNAU, Kunming, China), with the approval number 202310003.

### 2.2. Sample Collection and DNA Extraction

A total of 30 unrelated ZTPs were used in this study. Ear tissue samples were collected and soaked in 75% alcohol and stored in −80 °C freezers. Genomic DNA from ear tissues was extracted using a commercial kit (Tiangen Bio-chemical Technology Co., Ltd., Beijing, China) according to the manufacturer’s instructions. The quality of genomic DNA was checked by the Agilent 5400 analysis system (Agilent, Santa Clara, CA, USA) and 1% agarose gel electrophoresis.

### 2.3. Whole-Genome Sequencing and Data Collection

The genomic DNA sample was fragmented by sonication to reach a size of 350 bp. Then, the DNA fragments underwent end polishing, A-tailing, and ligation with the full-length adapter for sequencing, followed by PCR amplification. The PCR products were then purified using the AMPure XP system (Beckman Coulter, Beverly, MA, USA). Subsequently, the library quality was assessed using the Agilent 5400 system (Agilent, Santa Clara, CA, USA) and quantified by qPCR. The different libraries after quality control were pooled based on their effective concentration and the desired data amount. The 5′-end of each library was phosphorylated and cyclized. Finally, DNA sequencing libraries of all DNA samples were sequenced with an average depth of approximately 10× using the DNBSEQ-T7 platform (Novogene Bioinformatics Technology Co., Ltd., Beijing, China) to obtain 150 bp (PE150) paired-end raw reads. For comparison, 10 AWBs resequenced data sets were downloaded from the National Center for Biotechnology Information (NCBI) website (https://www.ncbi.nlm.nih.gov/, accessed on 10 June 2023). The accession numbers were SRR13630747, SRR13630748, SRR13630749, SRR13630750, SRR13630751, SRR13630752, SRR652378, SRR652379, ERR173220, and ERR173222.

### 2.4. Reads Mapping and SNP Detection

The raw reads were filtered to obtain clean reads by using fastp v0.23.4 software [11] with the default parameters. And the clean reads were mapped to the reference genome Sus scrofa11.1 by the index function of the BWA v0.7.18 software [12]. Then, alignment was performed using the MEM algorithm from BWA, and sorted binary bam files were obtained via picard. Next, picard tools were used to filter possible duplicate reads (REMOVE_DUPLICATES = true). The SNPs were detected using the Genome Analysis Toolkit (GATK, v3.6) [13]. Raw SNPs were detected using the “HaplotypeCaller”, “GenotypeGVCFs”, and “SelectVariants” modules of GATK, and then the sequencing and alignment artifacts were discarded from the SNPs by “VariantFiltration” with the parameters “QD < 2.0, MQ < 40.0, FS > 60.0, SOR > 3.0, MQRankSum < −12.5 and ReadPosRankSum < −8.0”. SNP annotation was performed using the ANNOVAR v2.0 software [14] with the parameters (-annotate_variation.pl -dbtype refGene). Finally, all the autosomal SNPs were filtered by using VCFtools v0.1.16 software [15] with the parameters “--min-alleles 2 --max-alleles 2 --maf 0.05 --max-missing 0.5”, and the genetic diversity, population structure, and selection signatures were analyzed based on the retained SNPs.

### 2.5. Genetic Diversity Analysis

The expected heterozygosity (H_E_), observed heterozygosity (H_O_), polymorphic marker ratio (P_N_), minor allele frequency (MAF), and inbreeding coefficient (Fis) were calculated using PLINK v1.9 [16]. In addition, π was calculated using VCFtools v0.1.16 software [15].

### 2.6. Population Structure Analysis

Originally, an NJ tree based on the distance matrix was constructed using the PHYLIP package [17] and visualized with the ggtree package [18]. After that, we pruned the SNPs in high levels of pair-wise LD using PLINK v1.90 [16] with the parameter (-indep-pair-wise 50 10 0.2) to perform PCA and ADMIXTURE analysis. PCA was performed by PLINK v1.90 [16], and the first two dimensions were used to distinguish population structure. Population structure analysis was based on all SNP information using ADMIXTURE v1.3.0 [19], and five-fold cross-validation was run to determine the cross-validation error minimum number of ancestral clusters (K). The K value was set from 1 to 5, and the ancestry compositions were visualized using the Pophelper package [20]. In addition, the genome-wide LD patterns of ZTPs and AWBs were assessed using PopLDdecay v3.42 software [21] with default parameters.

### 2.7. Genetic Distance and Relationship Analysis

The genetic distances based on identity by state (IBS) were calculated by PLINK v1.9 [16], and an IBS matrix was constructed to analyze the genetic distance between individuals within ZTPs. Meanwhile, a genomic relationship (G) matrix was constructed by GCTA v1.94 software [22] to analyze the genetic relationship between individuals within ZTPs. To make the numerical distribution more intuitive, the elements of the G matrix were normalized to the range of −1 to 1 and visualized using the pheatmap package in R v4.4.1 software.

### 2.8. Selection Signatures Analysis

In this study, selection signatures in ZTPs compared with AWBs were detected by two methods, F_ST_ and π ratio, using a 100 kb sliding window with a 10 kb step size in VCFtools v0.1.16 software [15]. According to the statistical results, only the overlapped window regions between F_ST_ and π ratio with the top 1% levels were considered as selected regions, where the candidate genes were detected. Then, candidate genes in the selected regions were annotated on the UCSC website (http://genome.ucsc.edu, accessed on 27 August 2023).

### 2.9. Functional Prediction Analysis

GO and KEGG pathways enrichment analyses were performed using clusterProfiler [23] and Pathview [24] packages, and the terms and pathways only exhibiting *p*-values < 0.05 were considered significant and listed.

## 3. Results

### 3.1. Sequencing and Detection of SNPs

A total of 1023.97 Gb of raw data was obtained for the 30 ZTP genomes; the average depth was 11.79×, and the summary statistics for the resequencing data were described in Table S1. Combined with the data of 10 AWBs from NCBI, a total of 45,514,452 autosomal SNPs were detected in the 40 pigs. The SNP annotation results indicated that these variations were most abundant in the intergenic region and least in the exonic region (Table S2). After filtering, 23,649,650 SNPs were retained to analyze the population structure and selection signatures.

### 3.2. Analysis of Genetic Diversity

ZTPs had the higher H_E_ (0.3271), P_N_ (0.9779), MAF (0.2380), π (0.3325), and lower H_O_ (0.2208) and Fis (0.0125) than AWBs (Table 1). Notably, the H_O_ was lower than H_E_ in the two populations.

### 3.3. Analysis of Population Structure

**Figure 1:**
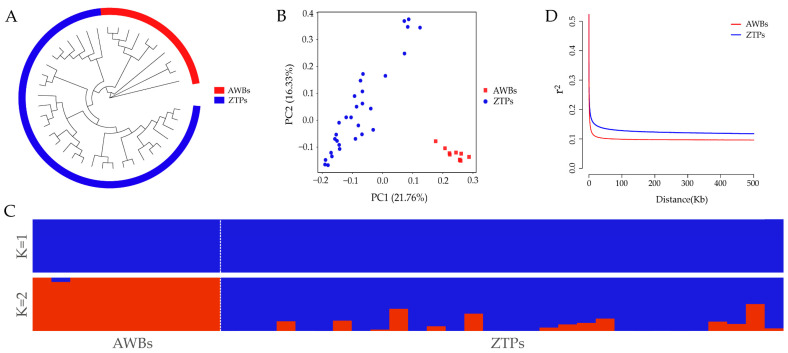
Population structure and LD analysis. (**A**) Neighbor-joining (NJ) tree. (**B**) Principal component (PC) plots for the first two PCs. (**C**) Structure analysis with K = 1 and K = 2. (**D**) Linkage disequilibrium (*r*^2^) decays of ZTPs and AWBs calculated over 50 kb windows.

It was shown that ZTPs and AWBs were two independent populations by NJ tree, PCA, and ADMIXTURE analysis. From the NJ tree, the two populations formed their own separate cluster (Figure 1A), but all individuals of ZTPs were divided into multiple branches. The result of PCA showed that ZTPs and AWBs were effectively separated, and 21.76% and 16.33% of the total genetic variation was explained by the first and second principal components, PC1 and PC2 (Figure 1B). In ZTPs, the distribution of some individuals was scattered and their relationships were distant. The degrees of mixture in the two populations with K = 2 (Figure 1C) further verified the results of the NJ tree and PCA. As shown in Figure 1D, with the increase in distance between SNPs, the overall decline trends of LD in the two populations were similar, but lower LD decay was observed in ZTPs than that in AWBs.

### 3.4. Genetic Distance and Relationship Between Individuals of ZTPs

**Figure 2:**
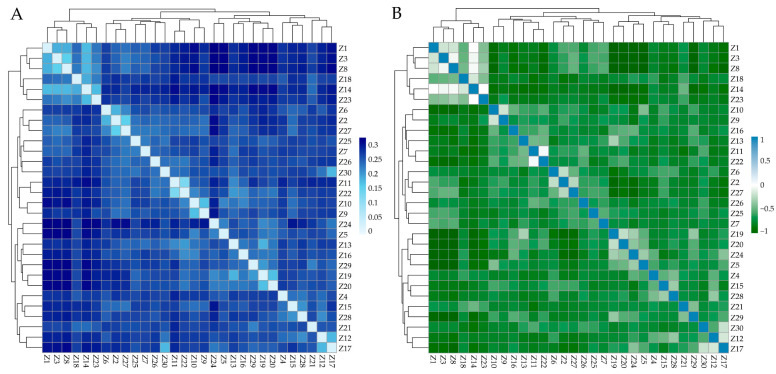
The heat map of IBS distance matrix and G matrix of ZTPs. (**A**) The IBS distance matrix of ZTPs. Each small square represents the genetic distance between the two individuals, and the color from light to dark indicates the genetic distance from low to high. (**B**) The G matrix of ZTPs. Each small square exhibits the value of the genetic relationship between the two individuals, which the colors blue and green from light to dark represent; the value ranges from 0 to 1 and 0 to −1, respectively.

The genetic distance between individuals in ZTPs ranged from 0.1321 to 0.3344, and the average genetic distance was 0.2708. The IBS genetic distance and G matrix of ZTPs showed that most of the individuals had large genetic distances (Figure 2A) and distant genetic relationships (Figure 2B). Additionally, all individuals of ZTPs were clustered into multiple branches.

### 3.5. Candidate Genes Under Selection Signatures

**Figure 3:**
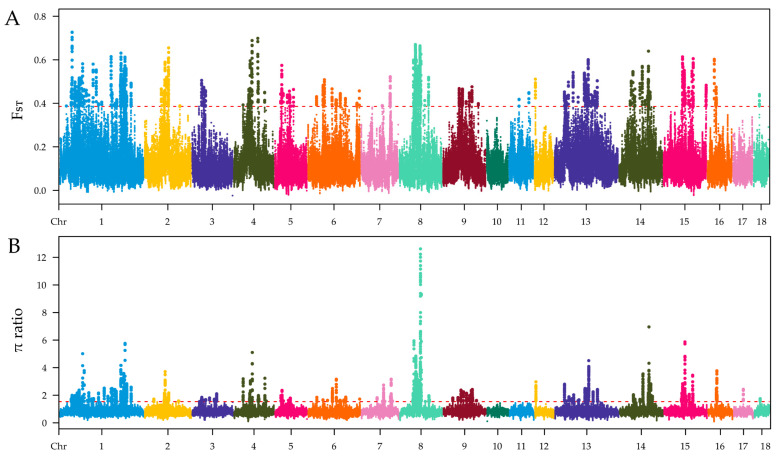
Identification of genomic regions with selection in ZTPs compared to AWBs, which are calculated in a 100 kb sliding window approach with 10 kb step size. (**A**) Distribution of F_ST_ values among autosomal chromosomes. (**B**) Distribution of π ratio among autosomal chromosomes. The red line represents the threshold value of F_ST_/π ratio.

**Figure 4:**
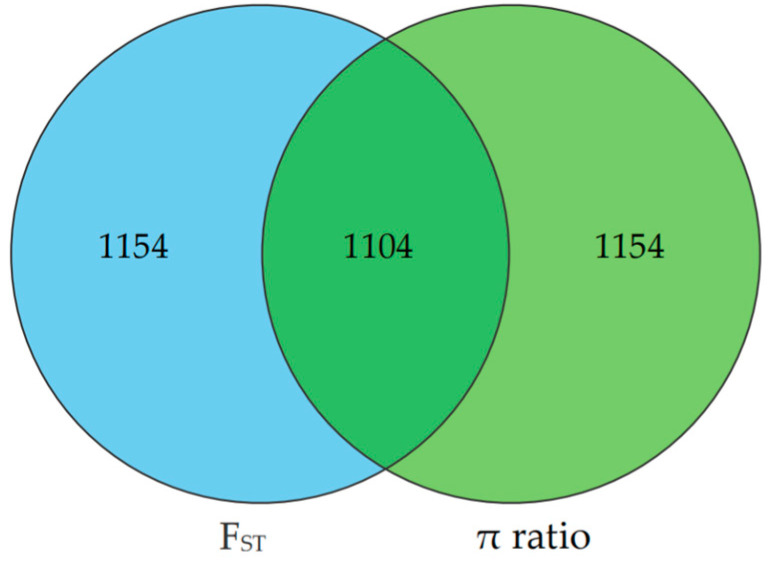
The Venn diagram shows the overlap in the number of candidate regions identified by F_ST_ and π ratio methods. The left and right circles represent the number of candidate regions identified by F_ST_ and π ratio approach, respectively.

The selection signatures in ZTPs were detected across the autosomes using F_ST_ and π ratio methods by comparing to those in AWBs. The Manhattan plot of the distribution of F_ST_ values and π ratio is shown in Figure 3A,B. A total of 2258 windows with the top 1% of F_ST_ value (F_ST_ ≥ 0.3859) were identified, which covered 42.10 Mb for 6.20% of the genome (Table S3). Based on the threshold of the top 1% π ratio (π ratio ≥ 1.5357), 2258 windows were also identified, covering 44.34 Mb for 6.53% of the genome (Table S4). Combined F_ST_ and π ratio approaches, totals of 1104 selected regions were identified (Figure 4 and Table S5). The selected regions were unevenly distributed across the genome. Most regions were observed on chromosomes (chr) 8 and 1 (381 and 322 regions, respectively), while no ones were found on chr 10, 11, and 17. Totals of 275 candidate genes were annotated within these selected regions (Table S6).

### 3.6. Functional Enrichment Analysis of Candidate Genes

GO analysis of the candidate genes showed that 59 genes were significantly enriched (*p*-value < 0.05) in 146 terms (Table S7), including 128 biological processes (BPs), 4 cellular components (CCs), and 14 molecular functions (MFs). And 23 genes were enriched in the top 10 GO terms with the smallest *p*-values (Table 2), including collagen fibril organization (GO:0030199), extracellular matrix structural constituent (GO:0005201), animal organ morphogenesis (GO:0009887), collagen trimer (GO:0005581), viral transcription (GO:0019083), regulation of viral transcription (GO:0046782), viral gene expression (GO:0019080), primary alcohol metabolic process (GO:0034308), response to amino acid (GO:0043200), and rough endoplasmic reticulum (GO:0005791). KEGG enrichment analysis showed that 20 genes were significantly enriched (*p*-value < 0.05) in seven pathways (Table 2), including sulfur metabolism (ssc00920), neuroactive ligand-receptor interaction (ssc04080), protein export (ssc03060), butanoate metabolism (ssc00650), ErbB signaling pathway (ssc04012), colorectal cancer (ssc05210), and MAPK signaling pathway (ssc04010).

Among the 36 genes enriched in the 10 most important GO terms and seven KEGG pathways (Table 2), 16 genes under selection might be associated with fat deposition (*NPY1R*, *NPY5R*, and *NMU*), reproduction (*COL3A1*, *COL5A2*, *GLRB*, *TAC3*, and *MAP3K12*), growth (*STAT6*, and *SQOR*), tooth development (*AMBN*, *ENAM,* and *ODAM*), and immune response (*MBL2*, *IL1A*, and *DNAJA3*).

## 4. Discussion

It is very important to investigate the genetic diversity, population structure, and selection signatures of indigenous pig breeds for understanding, evaluating, protecting, and utilizing these resources. In this study, whole genomes of 30 ZTPs were resequenced to analyze the genetic diversity, population structure, and selection signatures, combined with the genomic data from 10 AWBs. ZTPs had higher genetic diversity and lower inbreeding coefficients than AWBs. Compared with other Chinese indigenous pigs, the H_E_ of ZTPs (0.3271) was higher than that of Diannan small-ear pigs (0.2893) [25], Tongcheng pigs (0.31) [26], and Wuzhishan pigs (0.31) [27], but lower than that of Erhualian and Meishan pigs (0.38) [28], Liangshan pigs (0.35) [29], Min pigs (0.33) [30], etc. However, the H_O_ of ZTPs (0.2208) was lower compared to them (H_O_ ranged from 0.23 to 0.35) [25,26,27,28,29,30]. The NJ tree and PCA results clearly distinguished ZTPs from AWBs, which indicated that ZTPs had unique genetic characteristics as a result of long-term domestication and selection. However, 30 ZTPs were clustered into multiple branches; the distribution of the samples was scattered and accompanied by a low Fis (0.0125), which reflected the large genetic differences among individuals within the population. Meanwhile, the results of IBS genetic distances and the G matrix also showed that most of the individuals had large genetic distances and distant genetic relationships in ZTPs. It indicated that the selection of ZTPs needs to be further strengthened to improve the genetic uniformity. As domestic pig breeds, ZTPs exhibited a lower LD decay compared to AWBs, suggesting that artificial selection might have enhanced the LD degree in ZTPs.

Analyzing selection signatures of the genome can provide insights into the genetic mechanisms of pig adaptive phenotypes and identify important candidate genes related to excellent economic traits. ZTPs exhibit characteristics of unique body shape and appearance, young age of sexual maturity, high proportion of IMF, strong foraging ability, and adaptability owing to the domestication and selection for many years. Therefore, there should be some selection signatures on the ZTP genome. In this study, 1104 selected regions were identified by comparing ZTPs with AWBs, focusing on window regions that ranked in the top 1% for both F_ST_ values and π ratios. The largest number of selected regions was observed on chr 8, which is consistent with the previous study in Laiwu pigs [31], and they have 5.36 Mb (chr 8) overlapping regions. Compared with other studies in Chinese indigenous pigs, we also find 0.16 Mb (chr 1) overlapping regions in Wanbei pigs [5], and 0.72 Mb (chr 1) and 1.15 Mb (chr 8) overlapping regions in Anhui pig populations [7]. Within these selected regions, 275 candidate genes were further annotated. Functional enrichment analyses revealed that some candidate genes might play significant roles in fat deposition, reproduction, growth, tooth development, and immune response.

Among the candidate genes under selection, *NPY1R*, *NPY5R*, and *NMU* were potentially undergoing selection and were functionally associated with fat deposition. Neuropeptide Y (NPY) is a widely present neuro-hormone in the central and peripheral nervous systems, which plays a role in feeding behavior and energy balance [32]. Previous studies have suggested that *NPY1R* and *NPY5R* might be involved in mediating the orexigenic effects of NPY [33]. The selection signatures on *NPY1R* and *NPY5R* have been detected in some Chinese indigenous pig breeds, such as Tongcheng [34], Laiwu [31], and Anqing six-end-white [35] pigs, which showed that *NPY1R* and *NPY5R* were related to fat deposition in muscle of Laiwu pigs and positively selected in Anqing six-end-white pigs and involved in growth process. Neuromedin U (NMU) is a polymorphic neurotransmitter that regulates appetite and energy metabolism and is widely distributed in the gastrointestinal tract, hypothalamus, and pituitary [36]. NMU regulates body weight and energy balance in mice [37]. Transcriptomic analysis has shown that *NMU* is related to feed intake [38] and the growth and development of adipose tissue in broiler chickens [39]. As we know, ZTPs exhibit excellent meat quality, a high carcass fat percentage (>36%) [8], and an IMF content of longissimus dorsi (>6%) [9], which may be related to these genes under selection during the domestication and breeding.

Five selected genes associated with reproduction were detected, including *COL3A1*, *COL5A2*, *GLRB*, *TAC3*, and *MAP3K12*. *COL3A1* encodes the collagen alpha-1 (III) chain, which is an extracellular matrix protein found as a major structural component in hollow organs such as large blood vessels, uterus, and bowel [40]. The genomic organization and function of *COL5A2* were found to be similar to that of *COL3A1*, and both have evolved from a common ancestor gene [41]. A related study has shown that *COL3A1* and *COL5A2* were top hub genes related to the stage of serous ovarian cancers [42]. In animals, *COL5A2* may be a key candidate gene for uterine functional maintenance [43] and follicular development [44] in chickens. *GLRB* encodes the beta subunit of the glycine receptor, and research has shown that *GLRB* was highly expressed in the udder, kidney, uterus, and ovary in Dazu black goats, suggesting that it might affect reproductive traits [45]. *TAC3* encodes a neuropeptide protein (NKB) that is a member of the tachykinin family. Studies have shown that *TAC3* is related to the regulation of reproductive function in mice [46]. A distinctive finding indicates that the A63P variation in *TAC3* may increase the risk of earlier puberty onset in Chinese girls [47]. Additionally, *TAC3* may contribute to the sexual maturity in Xiang pigs [6]. *MAP3K12* is involved in the MAPK signaling pathway and is specifically selected in a high fecundity goat lineage, indicating it is associated with reproduction in dairy goats [48]. In pigs, *MAP3K12* has been identified as a reproduction-associated gene in Anhui indigenous pigs [7]. We infer that these genes may also have some association with the characteristics of young age of sexual maturity in ZTPs, but the exact effects still need to be studied and confirmed.

Two genes associated with growth, *STAT6* and *SQOR,* were identified in this study. *STAT6* is tightly linked to IL-4 and IL-13 signaling and plays a key role in the Th2 polarization of the immune system [49]. As a mediator of leptin signaling, *STAT6* may play an important role in the regulation of body weight by signaling the size of the adipose tissue mass [50]. It was reported that the polymorphisms of *STAT6* were associated with carcass and growth traits in feedlot cattle [51], and *STAT6* was one of the candidate genes underlying cattle growth QTL on chromosome 5 [52]. *SQOR* encodes the inner mitochondrial membrane in humans and plays an essential role in the catalysis and metabolism of hydrogen sulfide [53]. Recent studies have demonstrated that *SQOR* was associated with the growth and muscle development traits in Chinese Simmental beef cattle [54] and also with body size traits in Chinese Holstein cattle [55]. However, the effects of these two genes on the growth traits in pigs have not been reported.

Interestingly, three genes related to tooth development, *AMBN*, *ENAM*, and *ODAM*, were selected in ZTPs. Teeth are vital organs responsible for the survival and diversity of vertebrates due to their function in cutting, grinding, and crushing food, as well as their use in attack and defense [56]. Mammals have evolved complex and variable dentitions to adapt to the broad array of diets and environments [57]. Research has shown that the positive selection of tooth-related genes might promote the formation and bio-mineralization of tooth enamel and dentin, which would make the tooth structure stronger [58]. Ameloblastin (AMBN), enamelin (ENAM), and odontogenic ameloblast-associated (ODAM) are ameloblast-secreted proteins involved in various steps of the organization and mineralization of the enamel matrix of mammalian teeth [59]. Research has found that *AMBN* [60] and *ENAM* [61] genes played crucial roles in enamel formation, and *ODAM* was a major player in enamel mineralization and maturation [62]. In the past, ZTPs had been usually raised by grazing. It still maintains strong foraging ability till now. The genes related to tooth development were under selection, which further provides some indirect evidence for the strong foraging ability of ZTPs.

Moreover, some genes related to immune response, such as *MBL2*, *IL1A*, and *DNAJA3*, were also selected in ZTPs. Mannan-binding lectin (MBL) is a member of the innate immune system and plays an important role as the first line of host defense against certain infectious agents [63]. *MBL2* encodes the MBL-C protein, which participates in the humoral immune pathway by activating the complement effect and has anti-infective immune function in the host [64]. *IL1A* belongs to the interleukin-1 (IL-1) cytokine family, which is known to play a crucial role in immune cell activation and differentiation, as well as cell proliferation, maturation, migration, and adhesion, and may have pro-inflammatory, pro-fibrosis, or anti-inflammatory effects [65]. DNAJA3 (also called Tid1) is a member of the Hsp40 family of proteins and is known to play a significant role in the replication of several viruses [66,67]. In immune regulation, DNAJA3 regulated apoptotic resistance during activation-induced cell death of Th2 cells during T-cell activation, while DNAJA3 deficiency reduced expression of the anti-apoptotic *bcl-2* gene [68,69]. A recent study also suggested that DNAJA3 might be an immune regulator in B lymphocyte development [70]. It was widely known that ZTPs had been domesticated and raised in harsh environments and crude feeding conditions for a long time and formed strong adaptability and stress resistance. It is worthy to further study whether these genes are associated with the strong adaptability of ZTPs by regulating relative immune processes.

In summary, ZTPs had higher genetic diversity and a lower inbreeding coefficient compared to AWBs and exhibited a unique population structure and genetic differentiation within the population. The findings of selection signatures might provide novel insights into the roles of candidate genes in fat deposition, reproduction, growth, tooth development, and immune response of ZTPs.

## 5. Conclusions

This study revealed that ZTPs had a unique population structure, relative high genetic diversity, and obvious genetic differentiation within the population from the whole-genome sequence. A total of 1104 selected regions and 275 candidate genes were identified in ZTPs. Sixteen annotated genes were identified under selection that might be associated with specific characters, including three genes (*NPY1R*, *NPY5R*, and *NMU*) associated with fat deposition, five genes (*COL3A1*, *COL5A2*, *GLRB*, *TAC3*, and *MAP3K12*) with reproduction, two genes (*STAT6* and *SQOR*) with growth, three genes (*AMBN*, *ENAM*, and *ODAM*) with tooth development, and three genes (*MBL2*, *IL1A*, and *DNAJA3*) with immune response. These findings will contribute to the enhancement of conservation and breeding strategies for ZTPs and are of great significance for protecting and promoting the development of indigenous pig breeds in China.

## Figures and Tables

**Table 1:** The genetic variation of ZTPs and AWBs.

Populations	H_E_	H_O_	P_N_	MAF	π	Fis
ZTPs	0.3271	0.2208	0.9779	0.2380	0.3325	0.0125
AWBs	0.2805	0.2296	0.7912	0.2069	0.2967	0.1856

**Table 2:** The top 10 GO terms and seven KEGG pathways with the smallest *p*-values.

Terms/Pathways	*p*-Value	Genes
GO:0030199~collagen fibril organization	2.68483 × 10^−5^	*COL3A1*, *COL5A2*, *P4HA1*, *MMP11*
GO:0005201~extracellular matrix structural constituent	3.44971 × 10^−5^	*AMBN*, *ENAM*, *COL3A1*, *COL5A2*
GO:0009887~animal organ morphogenesis	0.0003	*NPY1R*, *NPY5R*, *AREG*, *AMBN*, *ENAM*, *NAB2*, *STAT6*, *COL3A1*, *COL5A2*, *SHOX2*, *ODAM*, *E2F5*
GO:0005581~collagen trimer	0.0007	*COL3A1*, *COL5A2*, *P4HA1*, *MBL2*
GO:0019083~viral transcription	0.0011	*REST*, *LOC100621006*, *TARBP2*
GO:0046782~regulation of viral transcription	0.0011	*REST*, *LOC100621006*, *TARBP2*
GO:0019080~viral gene expression	0.0018	*REST*, *LOC100621006*, *TARBP2*
GO:0034308~primary alcohol metabolic process	0.0018	*REST*, *LOC100621006*, *LOC100624541*
GO:0043200~response to amino acid	0.0021	*GLRB*, *COL3A1*, *COL5A2*
GO:0005791~rough endoplasmic reticulum	0.0024	*SEC63*, *RPS26*, *MBL2*, *SEC62*
ssc00920~sulfur metabolism	0.0035	*SUOX*, *SQOR*
ssc04080~neuroactive ligand-receptor interaction	0.0182	*NPY1R*, *NPY5R*, *GLRB*, *GRIA2*, *TAC3*, *RLN2*, *NPFF*, *NMU*
ssc03060~protein export	0.0182	*SEC63*, *SEC62*
ssc00650~butanoate metabolism	0.0214	*LOC110260333*, *L2HGDH*
ssc04012~ErbB signaling pathway	0.0407	*EREG*, *AREG*, *SOS2*
ssc05210~colorectal cancer	0.0470	*EREG*, *AREG*, *SOS2*
ssc04010~MAPK signaling pathway	0.0473	*EREG*, *AREG*, *IL1A*, *MAP3K12*, *MECOM*, *SOS2*

## Data Availability

The data presented in this study are available on request from the corresponding author.

## Supplementary Materials

The following supporting information can be downloaded at: https://www.mdpi.com/article/10.3390/ani14213129/s1, Table S1: The summary statistics for the resequencing data of ZTPs; Table S2: The result of variants annotation; Table S3: The information of the selected region for F_ST_; Table S4: The information of the selected region for π ratio; Table S5: The information of the 1104 selected regions; Table S6: The information of the 275 candidate genes; Table S7: GO analysis of the candidate genes.
